# Genetic and Morphometric Evidence for the Conspecific Status of the Bumble Bees, *Bombus melanopygus* and *Bombus edwardsii*

**DOI:** 10.1673/031.010.10901

**Published:** 2010-07-13

**Authors:** Robin E. Owen, Troy L. Whidden, R.C. Plowright

**Affiliations:** ^1^ Department of Chemical & Biological Sciences, Mount Royal University, 4825 Mount Royal Gate, S.W., Calgary, Alberta, Canada, T3E 6K6; ^2^ Department of Biological Sciences, University of Calgary, 2500 University Drive, NW, Calgary, Alberta, Canada, T2N 1N4; ^3^ Department of Zoology, Ramsay Wright Zoological Laboratories, 25 Harbord Street, University of Toronto, Toronto, Ontario, Canada, M5S 1A1; ^4^ Present address: Whidden Environmental Ltd., 17 Covepark Bay, NE, Calgary, Alberta, Canada T3K 6K8; ^5^ Present address: 108 Ch. River, Cantley, Quebec, J8V 3A1

**Keywords:** allozymes, color variation, taxonomy

## Abstract

The taxonomic status of closely related bumble bee species is often unclear. The relationship between the two nominate taxa, *Bombus melanopygus* Nylander (Hymenoptera: Apidae) and *Bombus edwardsii* Cresson (Hymenoptera: Apidae), was investigated using genetic (enzyme electrophoretic) and morphometric analyses. The taxa differ in the color of the abdominal terga two and three, being ferruginous in *B. melanopygus* and black in *B. edwardsii. B. edwardsii* occurs throughout California, while *B. melanopygus* extends north through Oregon, to Alaska and Canada. They are sympatric only in southern Oregon and northern California. The taxonomic status of these taxa was questioned when Owen and Plowright (1980) reared colonies from queens collected in the area of sympatry, and discovered that pile coloration was due to a single, biallelic Mendelian gene, with the red (*R*) allele dominant to the black (*r*). Here it is shown that all the taxa, whether from California, Oregon, or Alberta, have the same electrophoretic profile and cannot be reliably distinguished by wing morphometrics. This strongly supports the conclusion that *B. melanopygus* and *B. edwardsii* are conspecific and should be synonymized under the name *B. melanopygus*. Hence, there is a gene frequency cline running from north to south, where the red allele is completely replaced by the black allele over a distance of about 600 km.

## Introduction

The *Bombus* species (tribe Bombini) forms a well-defined monophyletic group containing a relatively small number of species (*n* = 239, according to Williams (1998)), thus it may seem surprising that *Bombus* species pose many taxonomic and systematic problems. At the supra-specific level, the conventional system of subgeneric divisions (Richards 1968) is fraught with inconsistencies, such as polyphyletic and paraphyletic subgenera (Williams 1998). Also, recent cladistic analysis suggests that it is no longer reasonable to retain the social parasite species in a separate genus, *Psithyrus*; only subgeneric status within *Bombus* is warranted (Williams 1995, 1998).

At the specific level the taxonomic status of closely related taxa is often unclear and subject to contradictory interpretations. *Bombus* species are relatively quite invariant or ‘monotonous’ morphologically compared to other bees (Michener 2000), but many species show considerable pile color variation. Some of this has a simple (Owen and Plowright 1980) or a relatively simple (Owen and Plowright 1988) genetic basis, but most variation is continuous and probably polygenic in nature (Stephen 1957). To complicate matters further, considerable convergence in color pattern, often between distantly related species, also occurs (Plowright and Owen 1980; Williams 2007). The bewildering amount of color variation led to an explosion of specific, sub-specific, and varietal names being applied in the early decades of *Bombus* taxonomy. Williams (1998) estimated that a maximum of 2800 different names were in use. Once the extent of this color variation was realized, taxa originally described as separate species by different authors were recognized to be the same, thus reducing the number of described species. The root of the problem is the limited number of traditional taxonomic approaches when applied to bumble bees. Genetic and statistical methods must be used to understand processes of speciation in *Bombus*. For example, Scholl et al. (1990) found that *Bombus moderatus* differed from *Bombus lucorum* at three out of 26 enzyme-gene loci, with the electromorphs exhibiting fixed differences in each species. In 1992, Scholl et al. (1992) found fixed electrophoretic differences between *Bombus auricomus* and *Bombus nevadensis* at five out of 18 enzyme loci. In both cases, the authors suggested the return to the original specific designations.

**Figure 1.**
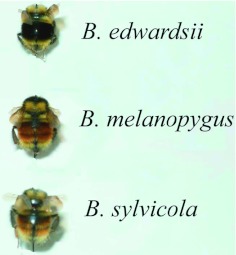
Typical specimens of the nominate forms of *Bombus edwardsii* Cresson, *Bombus. melanopygus* Nylander, and *Bombus sylvicola* Kirby (Hymenoptera: Apidae). High quality figures are available online.

**Figure 2.**
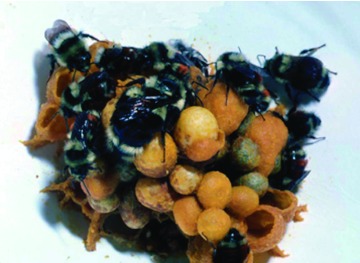
Colony number 27 raised by a queen collected in March 1979 from Smith River, California (see Figure 11 and Table 1 in Owen and Plowright, 1980). The genotype of the black queen is inferred to be *r/r* and she was mated to a red (*R*) male, thus all the workers in the colony are red and heterozygous. High quality figures are available online.

For this paper, the relationship between the two nominate taxa, *Bombus melanopygus* Nylander (Hymenoptera: Apidae) and *Bombus edwardsii* Cresson (Hymenoptera: Apidae), was examined in detail using a combination of genetic and morphometric analyses. Traditionally there was no question that these taxa represented two distinct species (Franklin 1913; Stephen 1957; Milliron 1961, 1971; Hurd 1979; Thorp et al. 1983). The bees differ dramatically in the color of the abdominal terga two and three (Figure 1); these being ferruginous (henceforth referred to as red) in *B. melanopygus* and black in *B. edwardsii*, although there are other morphological differences between the two (Table 1). Moreover, the distributions have relatively little overlap. *B. edwardsii* occurs throughout California and somewhat into neighboring Nevada (Hurd 1979; Thorp et al. 1983), while *B. melanopygus* extends north through Oregon, Washington, British Columbia, Alaska, east into Alberta, Saskatchewan, and across northern Canada possibly to Labrador (Stephen 1957; Hurd 1979; Curry 1984; Laverty and Harder 1988). They are sympatric only in southern Oregon and northern California (Stephen 1957; Thorp et al. 1983). However, the taxonomic status of these bees was called into question when Owen and Plowright (1980) reared colonies from queens collected in the area of sympatry. They discovered that pile coloration was due to a single, biallelic Mendelian gene with the red (*R*) allele dominant to the black (*r*); (see Figure 2). Also, the observed numbers of queen genotypes and colony types at each collection location conformed to those expected under Hardy-Weinberg equilibrium. This suggested that the two taxa are in fact conspecific and should be synonymized under the name *B. melanopygus* (as it has priority) with a gene frequency cline running from north to south where the red allele is completely replaced by the black allele over a distance of about 600 km (Owen and Plowright 1980; Owen 1986). Owen (1986) analyzed this cline theoretically and concluded that a selective differential on the order of 1% was sufficient to account for the observed transition. This genetic evidence is compelling (Williams 1998, 2008), but because the specimens were only collected from the region where both alleles are present, the logical possibility still exists that *B. edwardsii* is the dimorphic species and *B. melanopygus* exists as a separate, northern species.

**Table 1.**
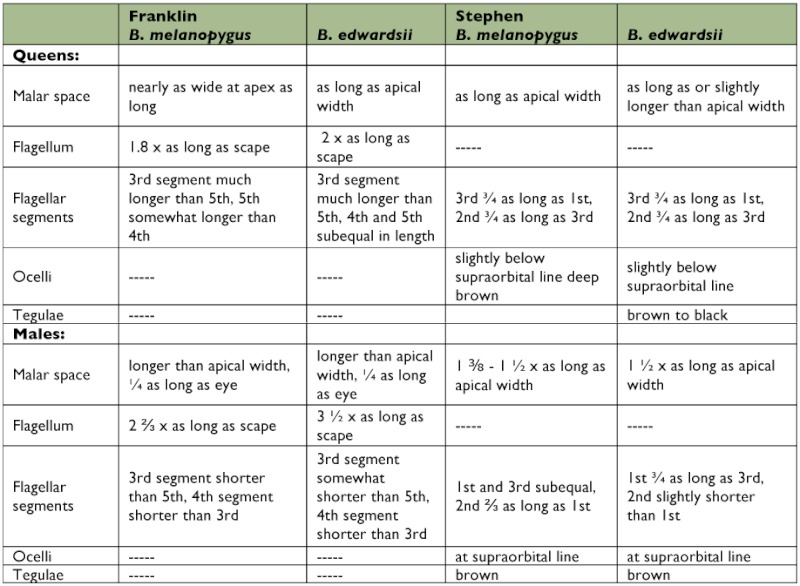
Descriptions of *Bombus melanopygus* and *B. edwardsii* taken from Franklin (1913) and Stephen (1957).

**Figure 3.**
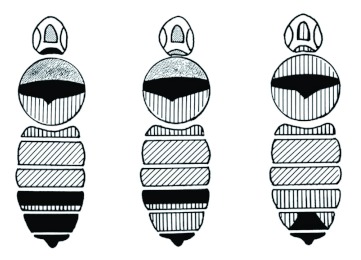
The color patterns of typical *Bombus melanopygus* (left), *Bombus sylvicola* (right), and the intermediate form (middle) as found in the Kananaskis River valley in Alberta. In the illustration, black = black pile on the bee, ferruginous (red) = crosshatching, yellow = vertical hatching, and admixture of yellow and black (which gives a “dusky” appearance) = stippling. Clear = bare. High quality figures are available online.

**Figure 4.**
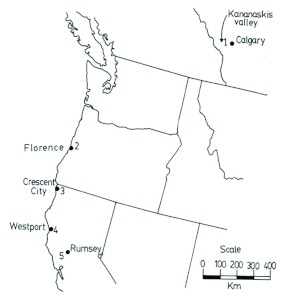
Locations where queens of the taxa were collected in 1988. Specimens of *Bombus edwardsii* and *Bombus melanopygus* (Hymenoptera: Apidae) were collected in California and Oregon from 13 February - 03 March. Specimens of B. *sylvicola* and *B. melanopygus* were collected in southern Alberta from 16 April - 16 June. High quality figures are available online.

Here, independent evidence is presented that these two taxa are indeed conspecific. Allozyme electrophoresis can be useful to distinguish closely related species. If there are fixed differences or a large gene frequency difference between two taxa, then this would strongly suggest either complete, or a very high degree of, reproductive isolation. Conversely, if two taxa have identical allozyme profiles, then this would strongly suggest conspecificity. Similarly, morphometric analysis of wing venation patterns has also proved to be very successful for differentiating between *Bombus* species (Plowright and Stephen 1973, 1980; Plowright and Pallet 1978). For this report, these techniques first are verified to be sensitive enough to correctly discriminate between *B. melanopygus* and a closely related species, *Bombus sylvicola* (Franklin 1913; Stephen 1957; Williams 2008) with which it is sympatric in Alberta. The relationship between these two species is, in itself, of considerable interest. The typical females (queens and workers) of the two species are easy to separate on the basis of color pattern (Figure 3), even though there are no other clear morphological differences (Stephen 1957). Also, the males of the two species have distinctly different genitalia. In *B. sylvicola* the apices of the penis valves are bulbous that is unique in North American *Bombus* (Thorp et al. 1983), whereas in *B. melanopygous* they are weakly pointed (Stephen 1957). Stephen (1957) noted that, in the interior valleys of British Columbia, females intermediate in color pattern occur and they are impossible to separate as to species; he speculated that they could be hybrids. In the Kananaskis Valley (Figure 4), which is just across the border from British Columbia, typical *B. melanopygus* forms tend to be found at lower elevations, while typical *B. sylvicola* forms and “intermediates” occur mostly at higher elevations. Altitudinal and other ecological differences also serve to separate the species to some extent (Hobbs 1967).

In this paper, it is shown that both enzyme electrophoresis and wing morphometrics do unambiguously distinguish between these two species. Even more interesting is that six specimens (queens) collected at high elevations and originally assigned by eye to *B. melanopygus* turned out to have the electrophoretic profile consistent with *B. sylvicola*. Moreover, they are grouped with *B. sylvicola* and clearly separated from *B. melanopygus* by wing morphometrics. This is particularly illuminating as this is “the exception that proves the rule” showing that color pattern is a poor predictor of relationship with these bees. Finally, it is shown that when the same analysis was applied to the *B. melanopygus/B. edwardsii* question, all the bees, whether from California, Oregon, or Alberta, have the same electrophoretic profile and cannot be reliably distinguished from each other by wing morphometrics.

The use of other characters has been purposely ignored in this analysis for two reasons. First, it is logically more secure to use characters that were not employed in the original descriptions of the species. Second, there appears to be little useful morphological variation with which to distinguish the species. The descriptions of the species from Franklin (1913) and Stephen (1957) have been extracted in Table 1. When compared directly, it is clear that the differences are more of a qualitative than a quantitative nature. The differences in the male genitalia, again, appear to be qualitative, and there is no clear distinguishing feature as with *B. melanopygus* and *B. sylvicola* (see Stephen 1957, for drawings). Indeed Franklin (1913, p. 338), when discussing *B. melanopygus*, goes so far as to state “The genitalia of the males of this species are very much like those of *fernaldi (sic), flavifrons, centralis* and *bimaculatus*.” (Note: Franklin mistakenly applied the name *B. fernaldi* to *B. edwardsii* (Stephen 1957)).

## Materials and Methods

### Bees

Queen bumble bees were collected in the spring of 1988 from the locations shown in Figure 4. Specimens were collected in California and Oregon from 13 February - 03 March. A total of 108 of the *B. edwardsii* form and 35 of the *B. melanopygus* form were collected. Most of these queens were installed in compact, mobile versions of the Plowright and Jay (1966) rearing box, 20 of these comprising a “bee hotel”. The rearing methods followed the procedures of Plowright and Jay (1966) as modified and described by Owen (2001). Any bees that died during the collection trip were pinned. All surviving specimens were transported back to the laboratory at the University of Calgary where colony rearing was continued. Eventually, any queens that had not started a colony were frozen at -70° C for subsequent electrophoresis. Queens heading colonies were permitted to live out their natural lifespan and then were pinned. All dead queens had their right forewing removed for the morphometric study. Specimens of *B. sylvicola* (35 queens) and *B. melanopygus* (39 queens) were collected in southern Alberta from 16 April - 16 June. Some of each species were installed for colony rearing; some were frozen for electrophoresis, and the rest were pinned. Ultimately, all had their right forewings removed for morphometrics.

### Electrophoresis

One hundred and thirteen (113) specimens were scored at 16 enzyme-gene loci (Table 2) using horizontal starch gel electrophoresis. The procedures and methods followed are given in Scholl et al. (1990) and Owen et al. (1992). The designation of electromorphs was standardized relative to the electromorph mobilities (in millimeters) of *B. occidentalis* (= index 100), so that the results reported here are comparable to those previously published for other species (Scholl et al. 1990).

### Wing morphometries

**Figure 5.**
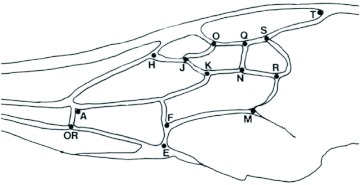
Right forewing of a *Bombus melanopygus* (Hymenoptera: Apidae) queen (Mel–08). The distance to point E from each of the other 13 points was measured. High quality figures are available online.

The technique was modified from Plowright and Stephen (1973) who measured the coordinates of 19 points on a wing by “…using the point OR as origin and the line OR-S as the horizontal axis” (Figure 5), which were standardized by dividing by the length of OR-S. The distances from E to the 13 points shown in Figure 5 were recorded. To do this, the right forewing of each bee was clamped between two glass microscope slides, then placed in a Kodak Trimlite F microfiche reader with a magnification of 24X. The distances were measured directly on the screen with a ruler to the nearest 0.5 mm.

**Table 2.**
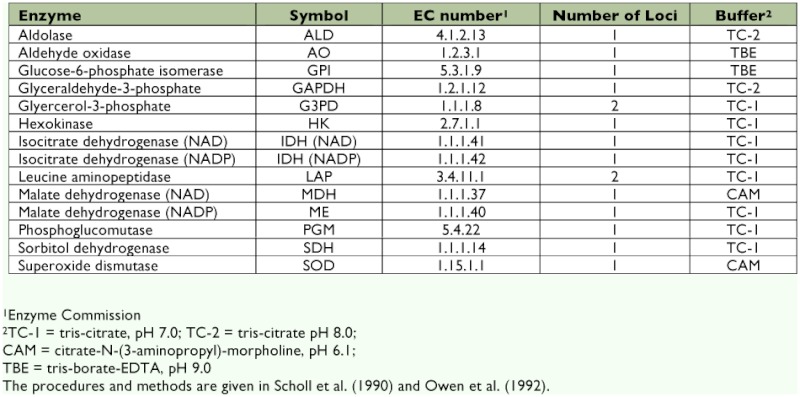
The list of the 14 enzymes stained with their Enzyme Commission numbers. A total of 16 enzyme-gene loci were scored as there were two loci discernible at both GAPDH and LAP.

Discriminant analysis was done using the statistical software package NCSS (Hintze 1996). Discriminant analysis was used to classify individuals into groups (Kachigan 1982; Hintze 1996). It derived prediction equations that maximized discrimination between groups; the goal was to be certain that individuals were placed in the preassigned groups according to a qualitative predictor variable. Mathematically, the technique is similar to multiple regression analysis, the difference being that in discriminant analysis the dependent variable is discrete instead of continuous (Hintze 1996). The predictor variable in this case was species name. In this case the null hypothesis was that the original classification of the taxa is correct, i.e. *B. melanopygus* and *B. edwardsii* are both distinct species. Since discriminant analysis derives equations that maximize distinction between groups it is an inherently conservative technique that was appropriate for these purposes, as this correspondingly minimized the likelihood of making a Type I error. Where real differences do exist the technique correctly discriminates between species (e.g. Plowright and Pallet 1978).

## Results

### Color dimorphism genetics

Only seven queens successfully established colonies. This low success rate was probably due to the continual movement from place to place. However the results (Table 3) confirm the findings of Owen and Plowright (1980) regarding the inheritance of the abdominal pile color dimorphism.

In Table 3, the male offspring are divided into the two categories: known worker-produced males and presumptive queen-produced males. *Bombus* species are haplodiploid, with males arising from unfertilized eggs and females from fertilized eggs. Since the workers are unmated they lay unfertilized eggs, which develop into males. The egg to adult development time in laboratory colonies of *B. melanopygus* is 23 days (Owen and Plowright 1980), thus any males eclosing 23 days after the queen's death must have been worker-produced.

**Figure 6.**
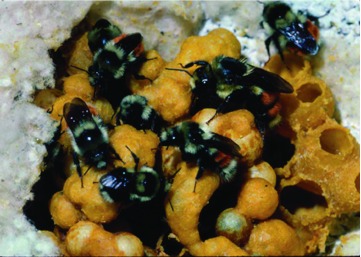
The colony raised by queen Mel–08 collected at Crescent City, California in March 1988 (see Figure 4 and Table 3). This red queen is inferred to be heterozygous (*R/r*) and mated to a black male (*r*). In this photograph the colony is at a fairly early stage of development and the coloration of only four red and one black worker can be seen clearly. High quality figures are available online.

In colonies headed by red queens, and producing red and black offspring (either females, males, or both), the queen must be heterozygous (*R/r*), and the expected ratio amongst the progeny produced by the queen is 1:1 red:black. This expectation was met in all four colonies of this type (Table 3, Figure 6). In three of these colonies workers produced males after the queen's death. If it is assumed that equal numbers of both worker genotypes lay eggs, then a 1:3 red:black ratio is expected. In colony Mel–08, there was a virtually perfect fit to this ratio, but this was not the case for the other two colonies in which there was a deficiency of black worker-produced males. This was only very slight in colony Mel–20, but was considerable in colony Mel–02 (Table 3).

**Table 3.**
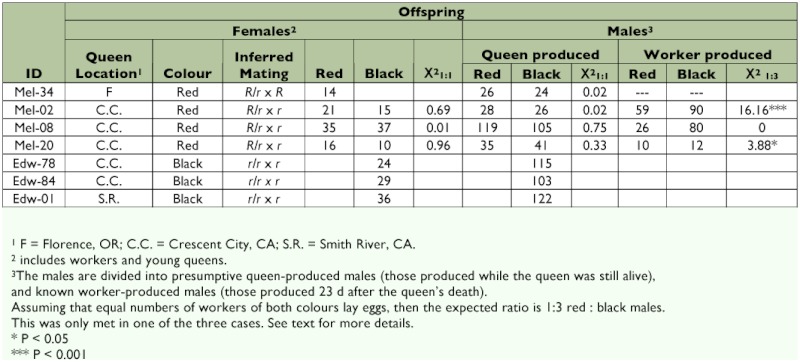
Progeny of queens collected in Oregon and California from February 13 to March 3, 1988.

### Electrophoresis

**Figure 7.**
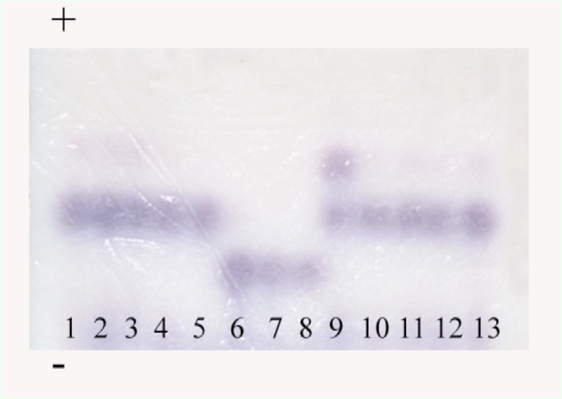
An example starch gel stained for phophoglucomutase (PGM). The gel was run from bottom (-) to top (+). Electromorph mobilities (*mm*) are standardized relative to those of *Bombus occidentalis* (Hymenoptera: Apidae) (= index 100, Scholl et al. 1990), and, from bottom to top, are 82, 93 and 100. Specimens 1 –5 are *Bombus melanopygus* from California, 6 – 8, *Bombus sylvicola* from Alberta, 9 – 13 *B. edwardsii* from California. Note that specimen 9 is heterozygous (also see Table 4). High quality figures are available online.

All bees had identical electrophoretic mobilities, and were invariant at 11 of the 16 enzyme loci examined. Five loci exhibited either differences between taxa and/or variation within taxa (Table 4). The nominate forms of *B. sylvicola* and *B. melanopygus* from Alberta (Figures 1, 2) clearly have different electrophoretic profiles (Table 4). Most significantly, there appear to be fixed differences at two enzyme loci: the characteristic electromorph for phosphoglucomutase (*Pgm*) in *B. sylvicola* is 82 (with one 72/82 heterozygote also detected), whereas in *B. melanopygus* the characteristic electromorph is 93 (see Figure 7). Similarly for hexokinase (*Hk*), the characteristic electromorphs are 105 and 100 for *B. sylvicola* and *B. melanopygus*, respectively. At sorbitol dehydrogenase (*Sdh*) 17 of the 18 *B. sylvicola* had electromorph 105, the other being 100, while all *B. melanopygus* had 100.

The predominant electromorph at isocitrate dehydrogenase (*Idh* — NAD) was 100 in *B. melanopygyus* and 95 in *B. sylvicola*; however, two of the latter were 100. Glucose-6phosphate dehydrogenase (*Gpi*) only showed variation in bees from Oregon and California, the characteristic electromorph being 96 in all taxa.

The electrophoretic profiles of *B. melanopygus* and *B. edwardsii* from all locations were entirely consistent with each other. There was a very small amount of variation present, with heterozygotes being detected at a few locations (Table 4). Overall, the average heterozygosity was low (*H*_exp_ = 0.031 ± 0.024), typical of most *Bombus* species (Owen et al. 1992). No heterozygotes were detected at the *Idh* (NAD) and *Sdh* loci, although presumptive homozygotes of different mobilities (100 and 102; 100 and 105 respectively) did occur. This may have been due to lack of resolution, allowing the bands to be interpreted as homozygotes (Owen et al. 1992). However, for the purposes of this investigation it is sufficient to regard the electromorphs as phenotypes.

There were six specimens (“MEL X” in Table 4) collected in Alberta that were assigned to *B. melanopygus* by eye when they were collected by REO, but turned out to have an electrophoretic profile inconsistent with that of *B. melanopygus* but consistent with that of *B. sylvicola*. Going back to the collection records it was found that these specimens (plus another three that were not electrophoresed) came from high elevations in the Kananaskis Valley (Fortress Mountain and Highwood Pass) where typical *B. sylvicola* had been collected. These were later reassigned to *B. sylvicola* on the basis of the wing morphometric analysis (see below).

**Table 4.**
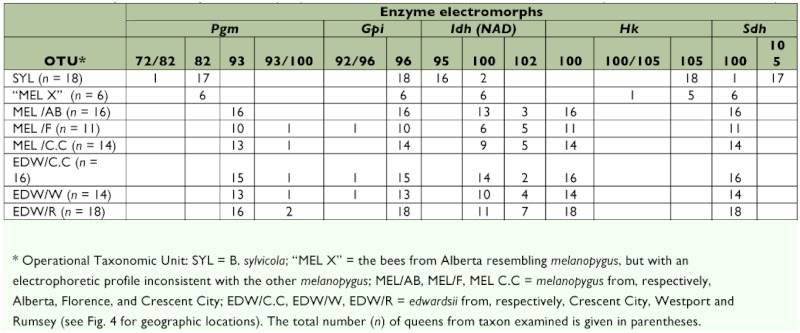
Electrophoresis results for the five enzymes exhibiting either differences between taxa and/or variation within taxa. The other 11 loci (Table 2) were invariant within, and showed no differences between, all taxa. The body of the table gives the number of individual bees of each electromorph. Electromorph mobilities (mm) are standardized relative to those of B. occidentalis (= index 100, Scholl et al. 1990).

### Wing morphometries

**Figure 8.**
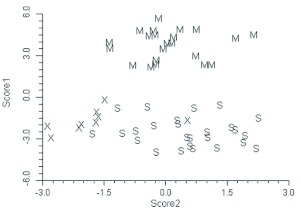
Plot of the first two Canonical scores for *Bombus sylvicola* (S), the Alberta *Bombus melanopygus* (M), and the anomalous Alberta *B. melanopygus* (“Mel X”) (Hymenoptera: Apidae). High quality figures are available online.

**Figure 9.**
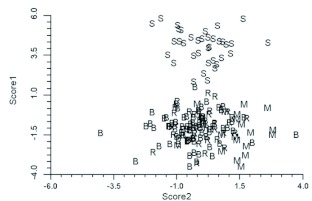
Plot of the first two Canonical scores for the total data set. S = *Bombus sylvicola* (Hymenoptera: Apidae), R = red *Bombus melanopygus* from Oregon and California, B = black *Bombus edwardsii* from Oregon and California, M = B. *melanopygus* from Alberta. High quality figures are available online.

**Figure 10.**
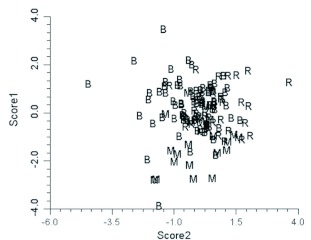
Plot of the first two Canonical scores for *Bombus melanopygus* and *Bombus edwardsii* (Hymenoptera: Apidae) only. R = red *B. melanopygus* from Oregon and California, B = black *B. edwardsii* from Oregon and California, M = *B. melanopygus* from Alberta. High quality figures are available online.

The discriminant functions analysis was run three times. Initially only specimens from Alberta were included. This was to verify that the technique could separate closely related species (*B. melanopygus* and *B. sylvicola*) in sympatry and to determine the status of the aberrant *B. melanopygus* (“MEL X”). In addition to the six “MEL X” bees that were electrophoresed (Table 4), three other queens that were collected on the same dates and at the same locations were reassigned from *B. melanopygus* and included in the “MEL X” category. The plot of the first two canonical scores is shown in Figure 8. *B. melanopygus* is clearly separated from *B. sylvicola* by the first canonical score. Similarly the “MEL X” specimens are obviously distinct from *B. melanopygus* and are grouped with *B. sylvicola*. Next, the analysis was run using the complete data set (Figure 9) with the “MEL X” specimens being reclassified as *B. sylvicola*. Again, *B. sylvicola* was clearly separated by canonical score one, but *B. melanopygus* and *B. edwardsii* were not obviously resolved. The classification count report (Table 5) showed that overall 34% of the *B. melanopygus* and *B. edwardsii* were misclassified, whereas none of the *B. sylvicola* were. Finally, only *B. melanopygus* and *B. edwardsii* were included (Figure 10, Table 6), which gave the same results, although the total percentage misclassified actually increased to 37%.

## Discussion

The results confirm the original genetic analysis of Owen and Plowright (1980); however as before, the interpretation is slightly complicated by the presence of worker-produced males. *Bombus* workers are effectively sterile, but possess ovaries that can undergo development if the workers are released from the dominance of the queen (Owen and Plowright 1980, 1982). This, of course, happens once the queen dies, but also occurs in some queenright colonies (Owen and Plowright 1982). The deviation from the expected ratio of worker-produced males in colony Mel-02 was probably because there were *not* equal numbers of red and black workers laying eggs. There are two reasons to believe this: first, in these colonies there was a preponderance of red workers (Table 3), and second, dominance hierarchies exist among egg-laying workers in bumble bee colonies (van Doom and Heringa 1986) with only some workers laying eggs at any given time (Owen and Plowright 1980). Thus it is quite reasonable to suppose that relatively more red workers contributed progeny than expected purely by chance. Owen and Plowright (1980) confirmed, in a similar case, by dissection that only some of the bees had developed ovaries.

**Table 5.**
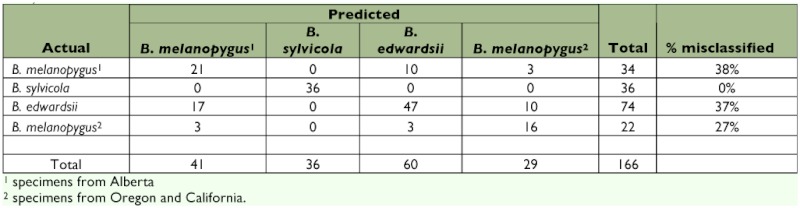
The classification count generated by the discriminant analysis on the total data set. This shows how accurately the discriminant functions classify the observations, and if classification is perfect then there will be zeros on the off-diagonals (Hintze 1996).

Enzyme electrophoresis and wing morphometries failed to distinguish the nominate species *B. edwardsii* and *B. melanopygus*, yet clearly separated *B. sylvicola* from the latter. This, together with the color dimorphism genetic data (Owen and Plowright 1980, and this paper), and the lack of other morphological differences (Table 1) strongly supports Owen and Plowright's (1980) view that *B. melanopygus* and *B. edwardsii* are conspecific, and should be named *B. melanopygus* (as this has priority).

**Figure 11.**
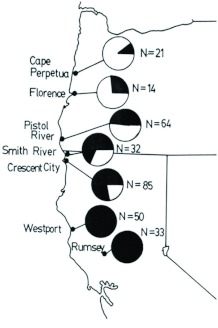
Gene frequency cline in *Bombus melanopygus* (Hymenoptera: Apidae) in Oregon and California. Pie diagrams give the relative frequency of the *R* (red) allele (clear portions) and the *r* (black) allele (shaded portions). The sample size (*N*) at each location represents the combined total of queen bees collected in 1978, 1979, 1980, 1981, and 1988. High quality figures are available online.

**Figure 12.**
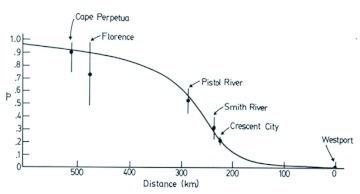
Frequency (*p*) of the dominant red (*R*) allele (± 95% confidence limits with sample sizes given in Figure 11) in queens of *Bombus melanopygus* (Hymenoptera: Apidae) along the coast of Oregon and California. The fitted line is the theoretical cline calculated using equations 29 and 30 of Owen (1986). High quality figures are available online.

If the proposition that these bees represent a single species is accepted, the question of intraspecific geographic variation, the most obvious being the color gene frequency cline, can be addressed. It follows that the cline has been generated *in situ* and results from a balance between selection and dispersal (Haldane 1948; Owen 1986) rather than representing a hybrid zone that would result from secondary contact of closely related species (Endler 1977; Barton and Hewitt 1985). Figures 11 and 12 show composite gene frequency estimates based on numbers of queen bees from Oregon and California collected on five separate trips from 1978–1988. Figure 12 also shows the theoretical curve for a cline derived for the X-linked or haplodiploid case by Owen (1986). The equations derived by Owen (1986) are the Xlinked versions of Haldane's (1948) equation for a cline with dominance. There is a very good fit between the observed data points and the theoretical curve. The possible selective pressure acting on this color dimorphism has been discussed elsewhere (Owen and Plowright 1980; Plowright and Owen 1980). The red morph appears to belong to one Müllerian mimicry group of bumble bees in the northwestern part of the continent while the black morph belongs to a corresponding one in California (Thorp et al. 1983).

**Figure 13.**
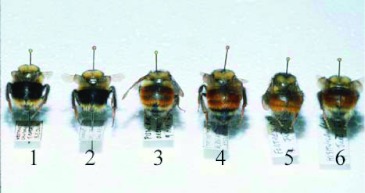
Clinal variation in tail (terga four and five) color in queens of *Bombus melanopygus* (Hymenoptera: Apidae) (specimens 1–5) as compared to *Bombus sylvicola* (specimen 6). Collection locations (also see Figures 3, 11, and 12): 1 = Westport (CA) , 2 = Crescent City (CA), 3 = Pistol River (OR), 4 = Cape Perpetua (OR) , 5 = Fortress Mountain (AB), 6 = Highwood Pass (AB). High quality figures are available online.

Treating *B. melanopygus* and *B. edwardsii* as conspecific now reveals somewhat parallel clinal variation in the amount of yellow pile on the tail (terga four and five) of queens, which gradually diminishes from south to north (Figure 13). It is most prominent in bees from Rumsey north to Crescent City, but in taxa near Cape Perpetua there is only a hint of yellow remaining. In Alberta the yellow is essentially absent, and is one feature that allows the typical *B. melanopygus* to be distinguished from the typical *B. sylvicola* (Figures 2, 13).

Similarly, the discriminant function analysis does suggest some geographic differentiation of *B. melanopygus* populations in wing morphology (Table 6). This not only reflects variation in shape (as described by the relative position of the points measured, see Figure 4), but also variation in the size of the bees since the measurements were not standardized (Figure 4) as done by Plowright and Stephen (1973). We deliberately did not do this for two reasons: one was to ensure that any differences between taxa would be maximized by the discriminant analysis, as pointed out earlier, the objective was to be as conservative as possible. The other reason was because size of *Bombus* queens is important ecologically (Owen 1988), and heritability of components of wing size has been demonstrated in other species (Owen 1988, 1989). Similarly, Owen and Harder (1995) found significant heritability for an allometric coefficient relating wing length and glossa length. There was also geographical variation of this coefficient in *B. vagans* (Owen and Harder 1995). Thus it is not surprising to find some geographic variation in such a widespread species as *B. melanopygus*. In the future, analyses of wing shape can proceed under the *a priori* assumption that intraspecific variation is being examined. The Procrustes method would be suitable, as this has been successfully used to analyze clinal variation of wing shape in the Australian *Drosophila serrata* (Hoffmann and Shirriffs 2002).

**Table 6.**
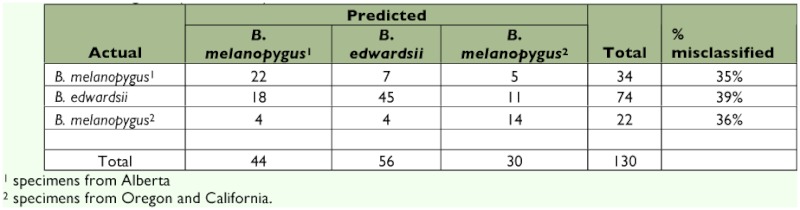
The classification count generated by the discriminant analysis using just the nominate *melanopygus* and *edwardsii*. This shows how accurately the discriminant functions classify the observations, and if classification is perfect then there will be zeros on the off-diagonals (Hintze 1996).

This study has also helped to clarify the relationship between *B. melanopygus* and *B. sylvicola* in Alberta, and there is no evidence for hybridization between the two as suggested by Stephen (1957). The queen specimens intermediate in color (Figure 2) are probably “pure” *B. sylvicola* and not hybrids. If the “MEL X” specimens (Table 4) were hybrids then they should be heterozygous at the gene loci with characteristic electromorph mobility differences between species (Table 4). Given the lack of heterozygotes at *Idh* and *Sdh* (see Results), this leaves *Pgm* and *Hk*. All six specimens had the *B. sylvicola* genotype at *Pgm* (82/82), and five of these also at *Hk* (105/105), the other being heterozygous (100/105). Since this bee obviously was homozygous at *Pgm* it is unlikely to be a hybrid because it should have been a heterozygote (82/93).

These results have wider applicability than just the elucidation of this taxonomic problem. They emphasize the necessity of using a combination of genetic and morphometric approaches to determine the relationship of *Bombus* taxa in general, and this has bearing on how species and sub-species in *Bombus* are defined. The acceptance of a single species (*B. melanopygus*) now allows geographical variation within this species to be seriously investigated. Of most interest is the color gene frequency cline. Gene frequencies (Figures 11 and 12) have only been estimated in queens, estimates from males are sorely needed. These are predicted to be the same as in females (Owen 1986), but because of the dominance of the red allele, the phenotypic frequencies are expected to differ between the sexes. Further investigation of the parallel clinal variation in the amount of yellow on the tail (Figure 13) is also warranted, as is more work on wing morphometric variation. All of this potentially makes *B. melanopygus* a model species for ecological genetic research.
